# Robot-assisted aspiration improves survival over conservative treatment in critical primary brainstem hemorrhage: a multicenter, volume-stratified cohort study

**DOI:** 10.3389/fneur.2026.1954969

**Published:** 2026-10-12

**Authors:** Haoming Guo, Xiaoliang Wang, Jialong Li, Shuoxuan Che, Qingyang You, Shang Gao, Lifeng Wu, Juncang Zhao, Zhiqiang Wang, Tao Li, Hailong Du, Xiaosong Liu, Lei Zhao, Xianming Wang, Shan Zhang, Wei Tian, Jianliang Wu

**Affiliations:** 1 Department of Neurosurgery, Hebei Children's Hospital, Shijiazhuang, China; 2 Department of Neurosurgery, The Second Hospital of Hebei Medical University, Shijiazhuang, China; 3 Hebei Medical University, Shijiazhuang, China; 4 Department of Neurosurgery, Jingxing County Hospital, Shijiazhuang, China; 5 Department of Neurosurgery, HanDan Central Hospital, HanDan, China; 6 Department of Neurosurgery, Handan First Hospital, HanDan, China; 7 Department of Neurosurgery, Affiliated Hospital of Hebei University of Engineering, HanDan, China

**Keywords:** hematoma volume stratification, minimally invasive surgery, primary brainstem hemorrhage, robot-assisted stereotactic aspiration, survival analysis

## Abstract

**Background:**

The optimal management of critical primary brainstem hemorrhage (PBSH, hematoma volume 5–15 mL) remains controversial. While minimally invasive surgery shows promise, multicenter evidence—particularly stratified by hematoma volume—is lacking. This study aimed to evaluate the survival benefit and safety of frameless robot-assisted aspiration compared to conservative treatment in patients with critical PBSH.

**Methods:**

We conducted a multicenter, retrospective cohort study involving 79 consecutive patients [Glasgow Coma Scale (GCS) 3–8] admitted to three neurosurgical centers between May 2022 and October 2025. Patients were allocated to a robot-assisted aspiration group (*n* = 38) or a conservative care group (*n* = 41). Further stratification was performed based on initial hematoma volume (5–10 mL, *n* = 63; >10 mL, *n* = 16). Volumetric measurements were performed by two independent neurosurgeons blinded to outcomes, demonstrating excellent inter-rater reliability (intraclass correlation coefficient = 0.96). Primary outcomes included 30-day and 90-day survival rates, analyzed using Kaplan-Meier curves and Cox proportional hazards regression. To rigorously control for potential treatment selection bias inherent in a retrospective cohort design, a 1:1 propensity score matching (PSM) sensitivity analysis was additionally performed.

**Results:**

Baseline characteristics, including age, GCS score, and hemorrhage location, were comparable between groups. Safety profiles, including rebleeding (7.89% vs. 4.88%) and intracranial infection rates, were similar (all *p* > 0.05). In the overall cohort, the robot-assisted aspiration group exhibited a significantly higher 30-day survival rate (76.3% vs. 43.9%, *p* = 0.006). Stratified analysis revealed that the survival benefit was confined to the 5–10 mL subgroup, where robot-assisted aspiration significantly improved 30-day (82.1% vs. 45.7%, *p* = 0.004) and 90-day survival (71.4% vs. 42.9%, *p* = 0.044), alongside improved discharge GCS scores. In the >10 mL subgroup (*n* = 16), 30-day survival was 60.0% (6/10) vs. 33.3% (2/6) — an absolute difference of +26.7 percentage points (95% CI: −19.9 to +59.8; Fisher's exact *p* = 0.61; *post-hoc* power 0.28). This subgroup was underpowered and the result is inconclusive rather than negative. Multivariable Cox regression confirmed robot-assisted aspiration as an independent protective factor (hazard ratio = 0.494, 95% CI: 0.245–0.997, *p* = 0.049), reducing mortality risk by 50.6%. In the PSM sensitivity cohort (34 matched pairs; 68 patients in total), robot-assisted aspiration still showed a significant survival benefit, reducing the risk of 90-day death by 60.0% (adjusted HR = 0.400, 95% CI: 0.195–0.822, *p* = 0.013). Volume stratification confirmed significant surgical benefits with respect to both survival and neurological function in the 5–10 mL subgroup (90-day survival rate: 73.1% vs. 32.1%, *p* = 0.003; distribution of mRS grades: *p* = 0.034), whereas the outcomes of the two treatment groups converged in the >10 mL subgroup (37.5% vs. 33.3%, *p* = 1.000).

**Conclusions:**

Robot-assisted aspiration is a safe intervention for critical PBSH and is associated with improved survival over conservative care — an association that persisted after propensity score matching — particularly within the 5–10 mL volume window. In hematomas larger than 10 mL the study was inconclusive: the point estimates favored aspiration but the subgroup was underpowered (*n* = 16; *n* = 14 after propensity score matching) and the confidence intervals included no benefit. Long-term functional outcomes were dominated by severe disability or death in both treatment arms regardless of volume. These findings suggest—rather than prove—a volume-dependent therapeutic window, underscoring the need for prospective validation to guide stratified neurocritical care.

## Introduction

Primary brainstem hemorrhage (PBSH) accounts for approximately 5% to 10% of all intracranial hemorrhages, yet it remains a formidable challenge in neurocritical care due to its extremely high mortality and disability rates (1). Currently, the prevailing international opinion still favors conservative management for brainstem hemorrhage, as rigorous conservative treatment can minimize iatrogenic injury (2). However, with increasing hematoma volume, the limitations of conservative therapy become progressively apparent. When the hematoma volume exceeds 5 mL, the early mortality rate of conservative treatment alone rises steeply. Multiple cohort studies have consistently shown that patients with hematoma volume >5 ml who receive only conservative treatment have a 30-day mortality rate generally ranging from 40% to 60%, and the long-term mortality rate remains persistently high at 70% to 80% (3–5). Evidently, under modern neurocritical care conditions, purely medical conservative management can no longer break the deadlock of high mortality in critical PBSH.

In recent years, frameless robotic-assisted neurosurgical systems, leveraging multimodal image fusion and submillimeter precision navigation, have significantly shortened preoperative preparation time, reduced surgical collateral damage, and improved 30-day survival rates, emerging as a promising interventional modality (6, 7). Nevertheless, controversy persists regarding the optimal hematoma volume range for which patients can truly benefit from this approach. It is widely accepted that patients with a hematoma volume < 5 mL are suitable for conservative treatment, whereas those with a volume >15 ml or presenting with deep coma upon admission often already have irreversible brainstem injury, leaving limited room for surgical salvage. Previous studies have indicated that when the hematoma volume crosses the threshold of 10 ml, the early mortality rate reaches an extremely dangerous peak (8). Based on this, we propose that 5–15 ml constitutes the key beneficial interval for minimally invasive surgery, and hypothesize that stratification at the 10 mL cutoff could more accurately reveal the true survival protective effect of robotic surgery across different severity levels. However, current clinical studies addressing this controversy mostly pool patients with 5–15 ml or even broader hematoma volumes together, generally lacking refined stratification based on specific hematoma volume. Consequently, the genuine differential efficacy of minimally invasive surgery across varying degrees of severity cannot be precisely identified.

Therefore, this multicenter stratified cohort study aims to clarify the value of robot-assisted puncture in patients with critical PBSH and hematoma volumes of 5–15 ml, elucidate the stratified survival differences across distinct hematoma volume grades, and verify whether this minimally invasive technique confers an independent survival protective effect in a multivariable model. The ultimate goal is to provide high-quality evidence-based medical evidence for stratified clinical decision-making and individualized minimally invasive intervention in critical PBSH.

## Materials and methods

This retrospective multicenter cohort study analyzed data from 79 consecutive patients with critical PBSH who met the inclusion and exclusion criteria and were admitted to the neurosurgery departments of the Second Hospital of Hebei Medical University, Handan Central Hospital, and Jingxing County Hospital between May 2022 and October 2025. All patients had a Glasgow Coma Scale (GCS) score of 3–8 at admission and a hematoma volume of 5–15 ml. Patients were divided into two groups according to the treatment strategy: the robot-assisted aspiration group (aspiration group, *n* = 38) and the conservative treatment group (conservative group, *n* = 41). Based on the initial hematoma volume, each group was further stratified into two subgroups: the 5–10 ml subgroup and the >10 ml subgroup.

### Inclusion and exclusion criteria

#### Inclusion criteria

①Imaging-confirmed primary brainstem hemorrhage with a hematoma volume of 5–15 ml.
②Admission GCS score of 3–8.
③Age ≥ 18 years.

#### Exclusion criteria

①Secondary brainstem hemorrhage caused by cerebrovascular malformation, aneurysm, tumor-related stroke, traumatic brain injury, or severe coagulation dysfunction.
②Concurrent multiple intracerebral hemorrhages or brainstem hemorrhage volume > 15 ml.
③Incomplete follow-up data.
④Presence of multiple underlying diseases or hepatic/renal insufficiency.

### Treatment protocols

#### Robot-assisted aspiration group

##### Target planning

Emergency thin-slice head computed tomography (CT) was performed before surgery and the images were imported into the workstation of the Neurosurgical Robot System for three-dimensional reconstruction of the hematoma. The optimal puncture trajectory was planned according to the shape of the hematoma, following the principles of shortest path and avoidance of critical vessels and nerve nuclei.

##### Registration and catheter placement

After successful general anesthesia, the patient was placed in the prone position and the head was fixed in a head frame. Two registration methods were used: !1! Skull-pin registration – spatial registration was achieved by identifying cranial bone markers with a registered probe, ensuring a registration error < 1 mm; !2! Structured-light registration – a structured-light scanner non-contactedly scanned the facial surface, and the point-cloud data were fused with the preoperative CT facial images for registration. After successful registration, a burr hole was drilled under the guidance of the robotic arm, the dura mater was punctured, and a drainage catheter was smoothly inserted into the predetermined target. Upon outflow of old blood, manual slow aspiration was performed, with the first aspiration volume controlled at 50%−60% of the initial hematoma volume. The catheter was then secured and connected to a closed drainage bag.

##### Postoperative liquefaction and catheter removal strategy

Head CT was repeated within 24 h after surgery to assess catheter tip position and residual hematoma. Once active bleeding was excluded, urokinase (30,000 IU dissolved in 3 ml saline, injected over > 1 min) was administered through the drainage tube, followed by clamping for 2 h and then opening for drainage, twice daily. Catheter removal was considered when follow-up CT showed residual hematoma < 2–3 ml, brainstem compression was relieved, and the drainage volume was < 5 ml/24 h. The indwelling time was typically 5–7 days.

#### Conservative treatment group

##### Management of cerebral edema and blood pressure

The patient's head was elevated to 30°. Dehydration and intracranial pressure reduction were achieved by combined administration of 20% mannitol (every 6–8 h), furosemide (10–20 mg), and albumin. Systolic blood pressure was strictly controlled between 120 and 140 mmHg using continuous infusion of urapidil or nicardipine.

##### Airway management

Immediate endotracheal intubation was performed if the admission GCS score was < 8 and SpO_2_ was < 90%. PaCO_2_ was maintained at 35–40 mmHg. Early tracheostomy was performed within 3–7 days after onset if ventilation support was anticipated to exceed 7–14 days.

##### Systemic support

Enteral nutrition was initiated early. Routine acid-suppressive therapy was given for 3–7 days to prevent stress ulcers. Lower-extremity pneumatic compression was applied to prevent deep vein thrombosis.

#### Data collection

The data collected and recorded in this study primarily comprised baseline characteristics, perioperative parameters, and follow-up prognostic indicators. Baseline characteristics included age, sex, history of hypertension and diabetes, hemorrhage location, admission GCS score, and initial hematoma volume measured by three-dimensional reconstruction using the Huake Precision Neurosurgical Robot System. To ensure the accuracy of volume-based stratification, two board-certified neurosurgeons, blinded to treatment allocation and clinical outcomes, independently performed volumetric measurements using the built-in software of the Huake Precision Neurosurgical Robot Workstation. Inter-rater reliability was excellent, with an intraclass correlation coefficient (ICC) of 0.96 (95% CI: 0.93–0.98). In cases where discrepancies exceeded 1 mL, a third senior neurosurgeon reviewed the images and adjudicated the final volume. Perioperative and surgical data included operative duration, intraoperative hematoma evacuation volume, residual hematoma volume on postoperative 24-h head CT, and various perioperative complications. Follow-up and outcome measures included GCS score at discharge, 30-day and 90-day mortality rates, and the 90-day modified Rankin Scale (mRS) score for assessing long-term neurological functional recovery. The final follow-up endpoint was 90 days after onset.

#### Statistical analysis

Statistical analyses were performed using SPSS version 29.0 and GraphPad Prism version 10.0. Continuous variables were tested for normality using the Shapiro–Wilk test. Normally distributed continuous variables are presented as mean ± standard deviation and were compared using the independent-samples *t*-test. Non-normally distributed continuous variables are presented as median (interquartile range) and were compared using the Mann–Whitney *U*-test. Categorical variables are expressed as frequencies (percentages) and were compared using the chi-square test or Fisher's exact test. Ordinal variables, including the distribution of 90-day modified Rankin Scale grades, were compared between groups using the Mann–Whitney U test. The 90-day post-onset period served as the follow-up endpoint. Cumulative survival curves were generated using the Kaplan–Meier method, and survival differences between groups were compared using the log-rank test. To control for baseline confounding and identify independent prognostic factors for 90-day survival, age and sex were forced into the multivariable Cox proportional hazards regression model (Enter method) regardless of their statistical significance in univariable analysis, together with variables with a univariable *P* value < 0.05. Hazard ratios (HR) and their 95% confidence intervals (CI) were calculated. All tests were two-sided, and a *p* < 0.05 was considered statistically significant. Given the retrospective, non-randomized cohort design of this study, 1:1 propensity score matching (PSM) was performed as a pre-specified sensitivity analysis in order to effectively mitigate the selection bias that may have been present in treatment allocation. Propensity scores were estimated using a multivariable logistic regression model, and the covariates entered into the model covered the key baseline confounders: age, sex, admission GCS score, initial hematoma volume, and hemorrhage location. Nearest-neighbor matching without replacement was applied, with the caliper width set at 0.2 standard deviations of the logit of the propensity score. In the successfully matched cohort, between-group baseline balance, the main stratified efficacy outcomes (GCS score at discharge, 30-day and 90-day survival rates, and the distribution of 90-day mRS scores), and the multivariable Cox proportional hazards regression model were re-evaluated, in order to verify the robustness of the principal study conclusions after removal of selection bias.

## Results

### Baseline characteristics

No statistically significant differences were observed between the aspiration group and the conservative group in terms of age, sex, history of hypertension and diabetes, admission GCS score, or hemorrhage location, either in the overall cohort or in any of the hematoma volume subgroups (all *p* > 0.05), confirming the comparability of the two groups (Table 1).

**Table 1:** Baseline characteristics of patients with critical pbsh undergoing robot - assisted aspiration or conservative treatment, stratified by hematoma volume.

Characteristic	Overall (*n* = 79)			5–10 ml subgroup (*n* = 63)			>10 ml subgroup (*n* = 16)		
	Conservative group (*n* = 41)	Aspiration group (*n* = 38)	*p*-value	Conservative group (*n* = 35)	Aspiration group (*n* = 28)	*p*-value	Conservative group (*n* = 6)	Aspiration group (*n* = 10)	*p*-value
Age (years, x ± s)	52.8 ± 13.7	49.9 ± 11.8	0.314	51.7 ± 14.1	50.5 ± 11.5	0.710	58.8 ± 9.9	48.0 ± 13.0	0.084
Sex (male), *n* (%)	34 (82.9)	28 (73.7)	0.414	29 (82.9)	19 (67.9)	0.235	5 (83.3)	9 (90.0)	1.000
Hypertension (yes), *n* (%)	30 (73.2)	29 (76.3)	0.800	24 (68.6)	22 (78.6)	0.408	6 (100.0)	7 (70.0)	0.250
Diabetes (yes), *n* (%)	8 (19.5)	4 (10.5)	0.353	6 (17.1)	3 (10.7)	0.719	2 (33.3)	1 (10.0)	0.518
Admission GCS score (points, x ± s)	3.8 ± 1.3	4.3 ± 1.7	0.156	3.9 ± 1.4	4.6 ± 1.8	0.122	3.2 ± 0.4	3.5 ± 0.5	0.181
Hemorrhage volume (mL, x ± s)	8.0 ± 2.5	8.3 ± 3.0	0.575	7.2 ± 1.7	6.7 ± 1.4	0.229	12.3 ± 1.5	12.7 ± 1.7	0.645
Hemorrhage location, *n* (%)	—	—	0.646	—	—	0.711	—	—	0.726
Pons	31 (75.6)	26 (68.4)		27 (77.1)	20 (71.4)		4 (66.7)	6 (60.0)	
Pons + Midbrain	9 (22.0)	9 (23.7)		7 (20.0)	6 (21.4)		2 (33.3)	3 (30.0)	
Midbrain	1 (2.4)	2 (5.3)		1 (2.9)	2 (7.1)				
Pons + Medulla oblongata	0 (0.0)	1 (2.6)					0 (0.0)	1 (10.0)	

Data are presented as mean ± SD (for continuous variables) or *n* (%) (for categorical variables). GCS, Glasgow Coma Scale; PBSH, Primary brainstem hemorrhage. *p*- values were calculated using independent *t*- test (continuous variables) or χ^2^ test (categorical variables).

### Safety outcomes

There were no significant differences between the two groups in the rates of postoperative rebleeding, intracranial infection, perioperative pneumonia, or stress ulcer (all *p* > 0.05) (Table 2).

**Table 2:** Perioperative complications in patients with critical PBSH undergoing conservative treatment or robot - assisted aspiration.

Complication	Conservative group (*n* = 41)	Aspiration group (*n* = 38)	*p*-value
Rebleeding	2 (4.88)	3 (7.89)	0.667
Intracranial infection	1 (2.44)	1 (2.63)	1.000
Pneumonia	32 (78.0)	28 (73.7)	0.793
Stress ulcer	11 (26.8)	12 (31.6)	0.805

Data are presented as *n* (%). *p*- values were calculated using χ^2^ test for categorical variables.

### Neurological improvement (GCS score)

In the overall cohort, the aspiration group exhibited a greater improvement in GCS score from admission to discharge compared with the conservative group. In the 5–10 ml subgroup, the discharge GCS score was significantly higher in the aspiration group than in the conservative group. In the >10 ml subgroup, neither group showed substantial improvement in GCS score at discharge (Figure 1).

**Figure 1:**
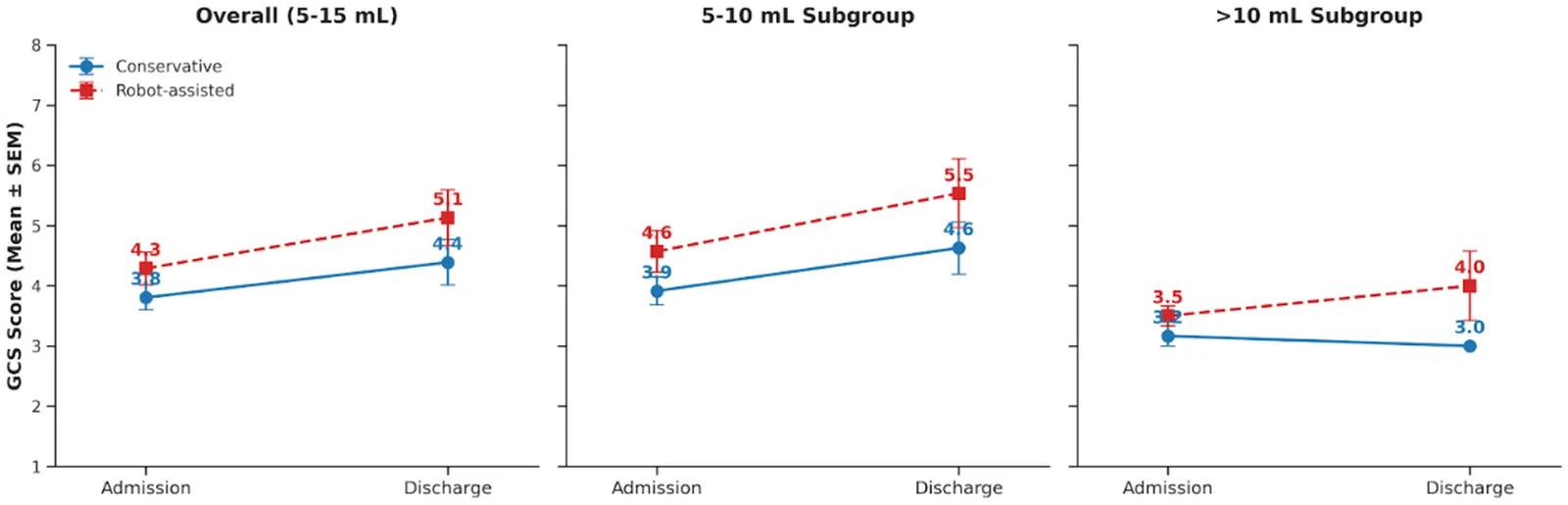
Changes in GCS scores from admission to discharge in total population and hematoma volume subgroups.

### 30 -day survival rate

The 30-day survival rate was significantly higher in the aspiration group than in the conservative group in both the overall cohort (76.3% vs. 43.9%, *P* = 0.006) and the 5–10 ml subgroup (82.1% vs. 45.7%, *p* = 0.004). In the >10 mL subgroup, 30-day survival was 60.0% (6/10) vs. 33.3% (2/6), an absolute difference of +26.7 percentage points (95% CI: −19.9 to +59.8; Fisher's exact *p* = 0.61). Because this stratum comprised only 16 patients, the comparison had a *post-hoc* power of 0.28 and is reported as inconclusive; detecting a difference of this magnitude at α = 0.05 with 80% power would require approximately 60 patients per group (Figure 2).

**Figure 2:**
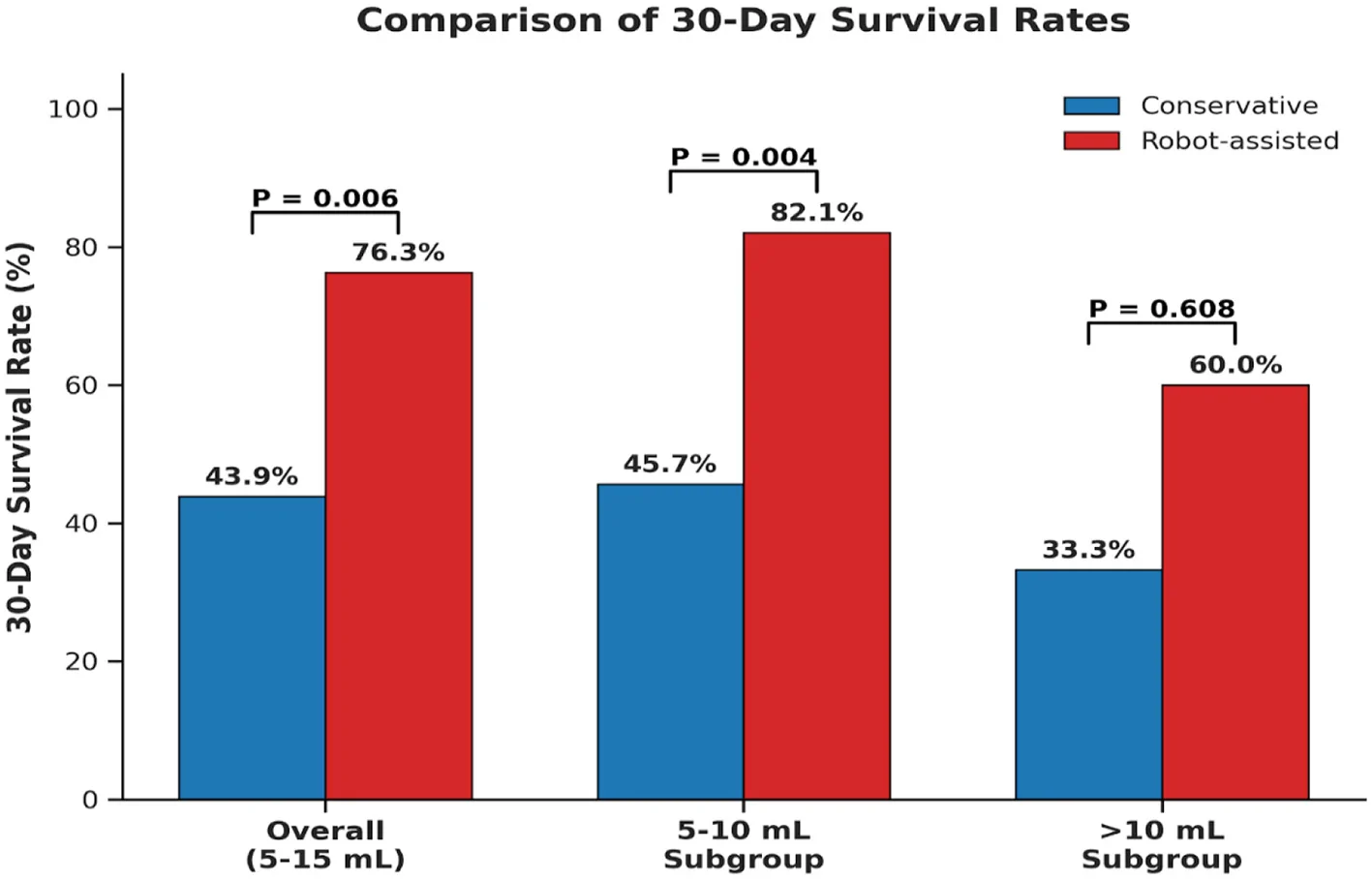
Comparison of 30-day survival rates between robot-assisted aspiration and conservative treatment for critical PBSH stratified by hematoma volume.

### 90 -day survival rate

In the 5–10 ml subgroup, the 90-day survival rate was significantly higher in the aspiration group than in the conservative group (71.43% vs. 42.86%, *p* = 0.044). In the overall cohort (63.2% vs. 41.5%, *p* = 0.089) and in the >10 mL subgroup (40.0% [4/10] vs. 33.3% [2/6]; absolute difference +6.7 percentage points, 95% CI: −36.7 to +43.9; Fisher's exact *p* = 1.000), the aspiration group showed numerically higher survival rates. In the >10 mL subgroup this result should be regarded as inconclusive rather than as evidence of no difference (Figure 3).

**Figure 3:**
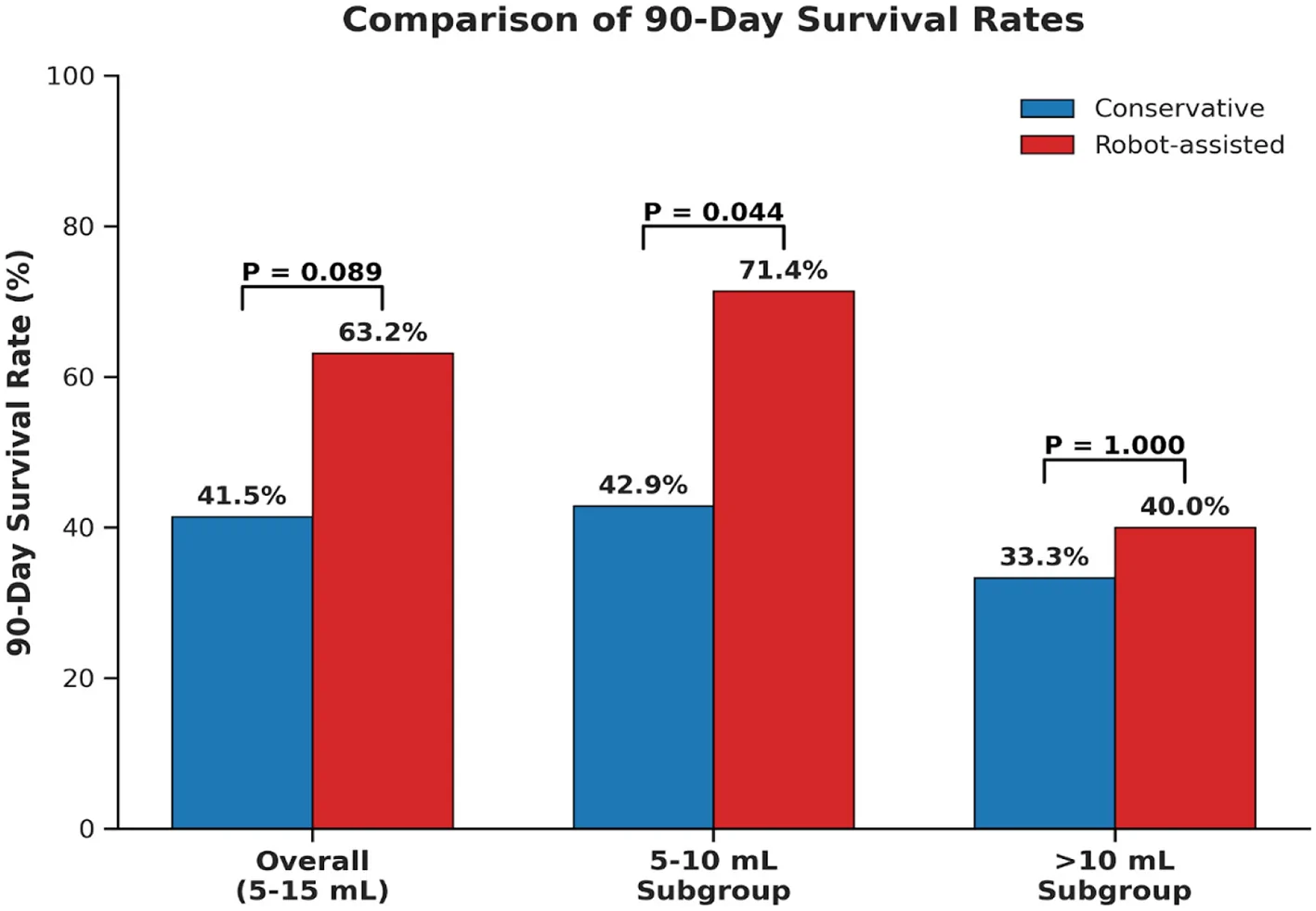
Comparison of 90-day survival rates between robot-assisted aspiration and conservative treatment for critical PBSH stratified by hematoma volume.

### Kaplan–Meier survival analysis

In the overall cohort and the 5–10 ml subgroup, the Kaplan–Meier survival curves of the two groups began to diverge markedly within the first 14 days after onset. The survival rate in the conservative group declined more rapidly during the first two weeks, whereas the curve for the aspiration group showed a significantly slower decline and entered a stable plateau phase after approximately 20 days. In the >10 mL subgroup (exploratory; 10 and 6 patients), both curves declined steeply within the first 14 days; thereafter, the aspiration group entered a relatively gradual phase, and the long-term survival curves of the two groups eventually converged. With only 16 patients and 10 events in this stratum, the shape of these curves should not be over-interpreted (Figure 4).

**Figure 4:**
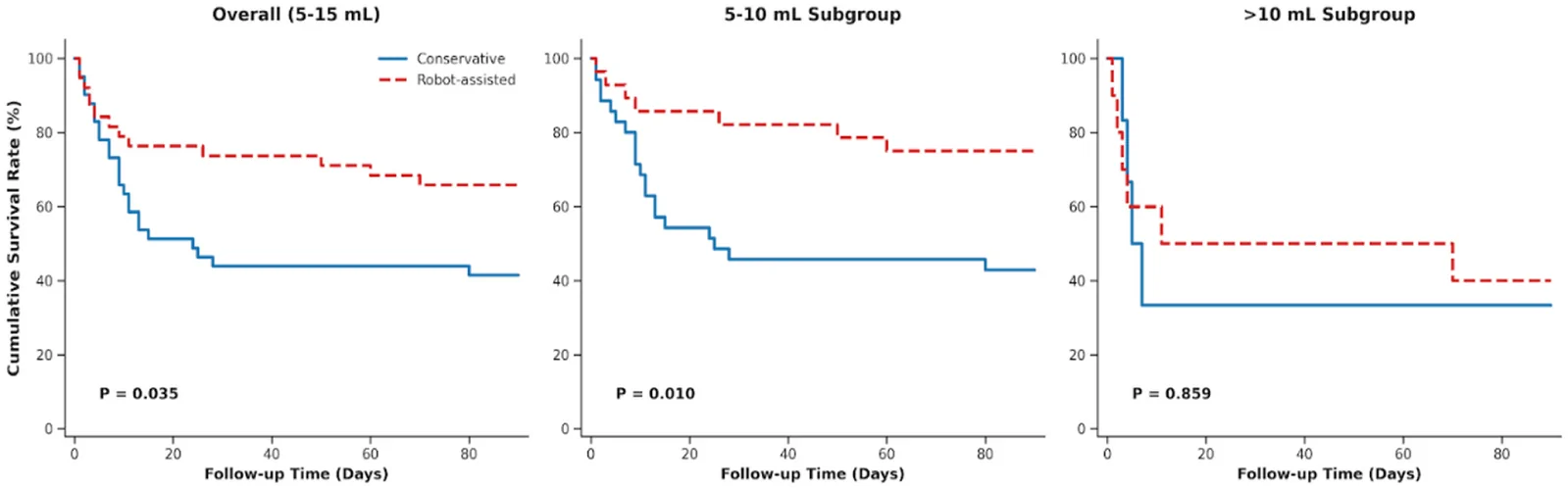
Kaplan–Meier survival analysis: robot-assisted aspiration vs. conservative treatment stratified by hematoma volume.

### Functional outcome (mRS score at 90 days)

Compared with the conservative group, the aspiration group showed a lower proportion of patients with mRS score 6 (death) and a correspondingly higher proportion of patients with mRS score 5 (severe disability). In the >10 mL subgroup, only 4 of 10 patients in the aspiration group and 2 of 6 in the conservative group were alive at 90 days, and every survivor in both arms had an mRS grade of 5 (severe disability); no patient in this stratum achieved functional independence (Figure 5). This pattern indicates that the survival advantage was absorbed almost entirely by the mRS grade 5 category: patients who would otherwise have died instead survived with severe disability requiring continuous care, rather than achieving functional independence or moderate disability. The proportion of patients free of severe disability or death was therefore not demonstrably increased.

**Figure 5:**
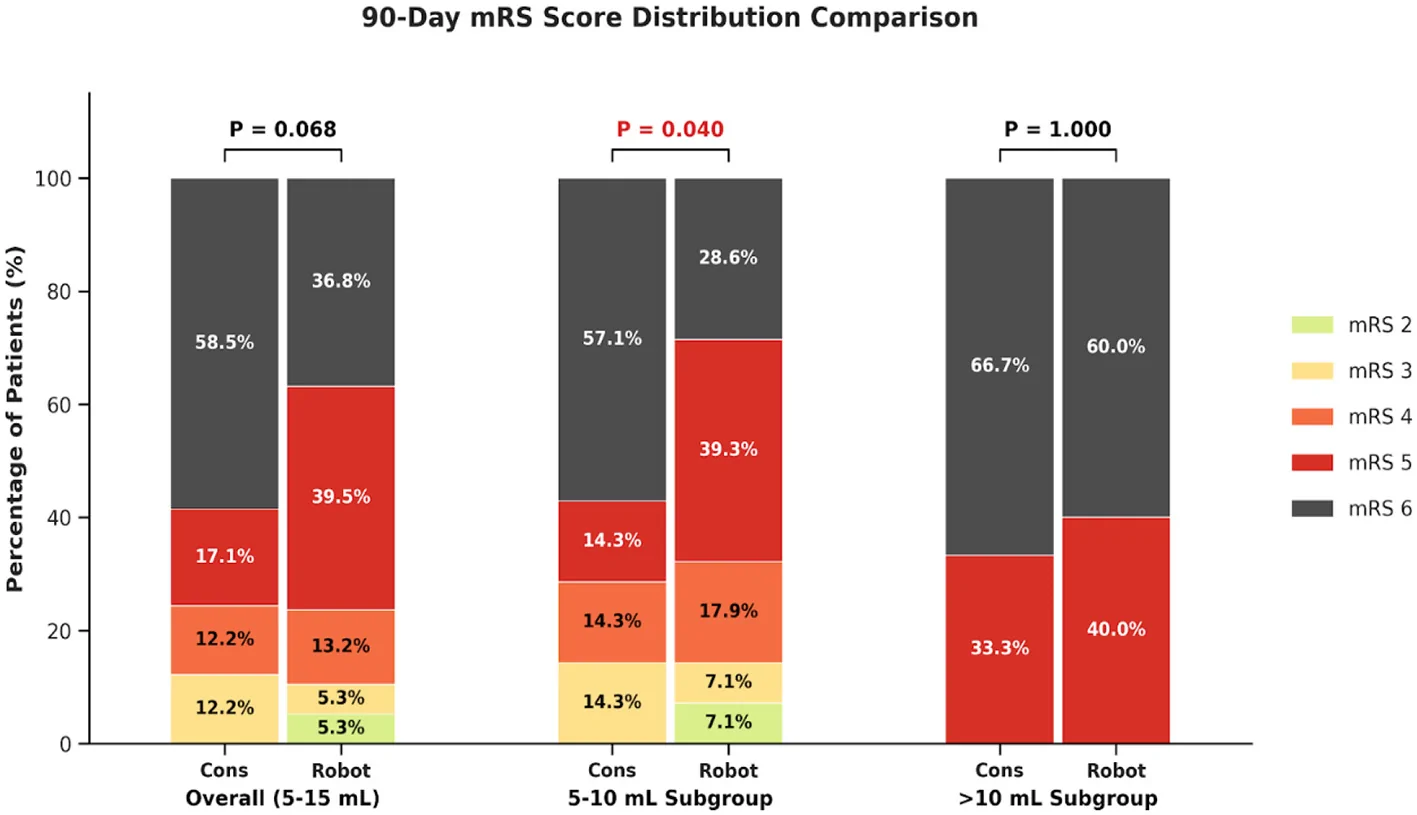
Comparison of 90-day mRS score distribution between robot-assisted aspiration and conservative treatment for critical PBSH stratified by hematoma volume.

### Cox proportional hazards regression analysis for 90-day survival

Univariable Cox regression analysis revealed that admission GCS score (HR = 0.713, *p* = 0.020), initial hematoma volume (HR = 1.165, *p* = 0.004), and treatment modality (HR = 0.494, *p* = 0.041) were significantly associated with 90-day survival, whereas age, sex, history of hypertension, and history of diabetes were not (all *p* > 0.05) (Figure 6).

**Figure 6:**
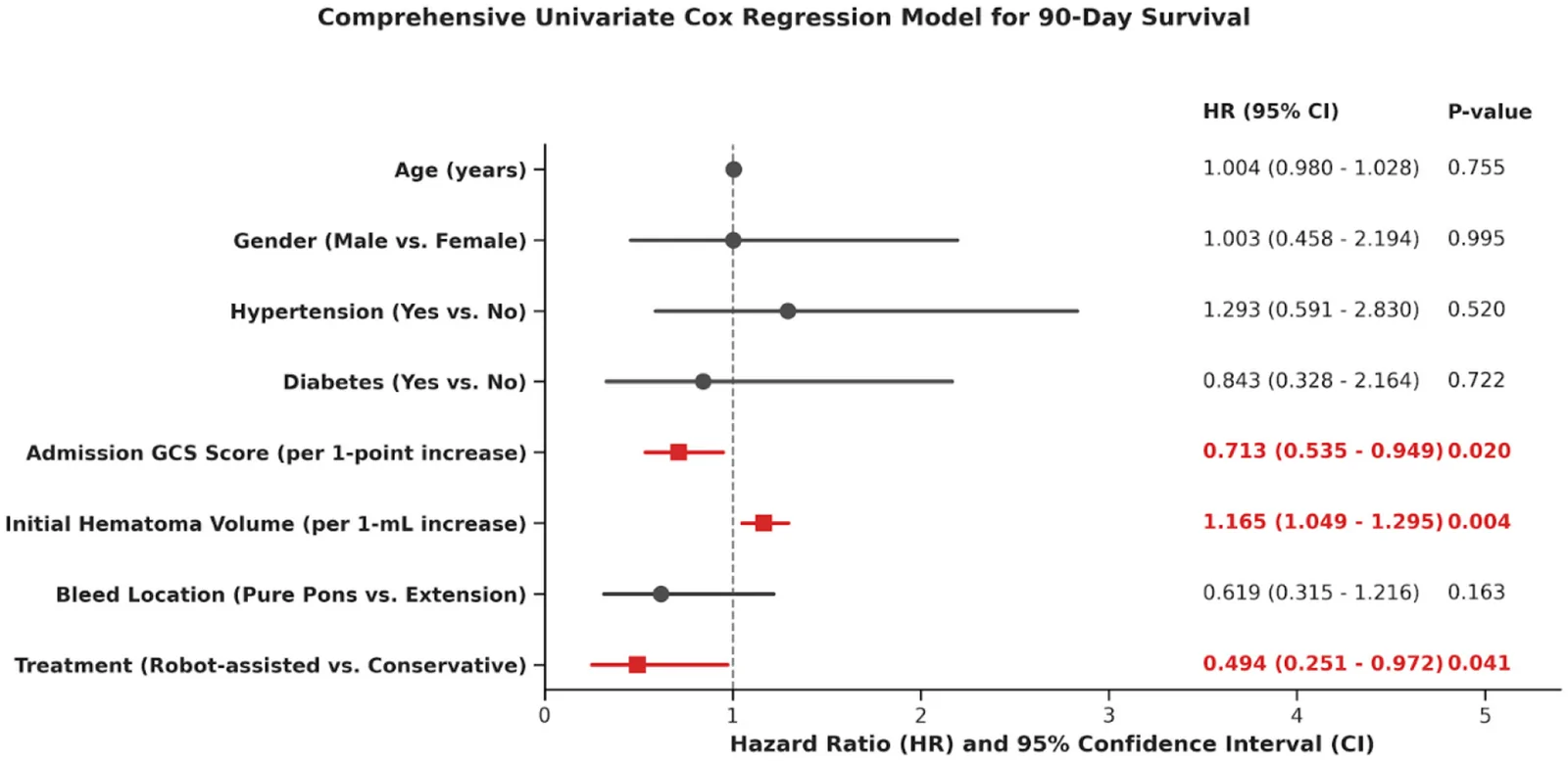
Univariable Cox regression analysis for 90-day survival of critical PBSH patients (Forest plot).

After adjustment in the multivariable Cox model, initial hematoma volume remained an independent risk factor for 90-day mortality (HR = 1.173 per 1ml increase, 95% CI: 1.039–1.325, *p* = 0.010), while robot-assisted aspiration was confirmed as a significant independent protective factor, reducing the risk of death by 50.6% compared with conservative treatment (HR = 0.494, 95% CI: 0.245–0.997, *p* = 0.049) (Figure 7).

**Figure 7:**
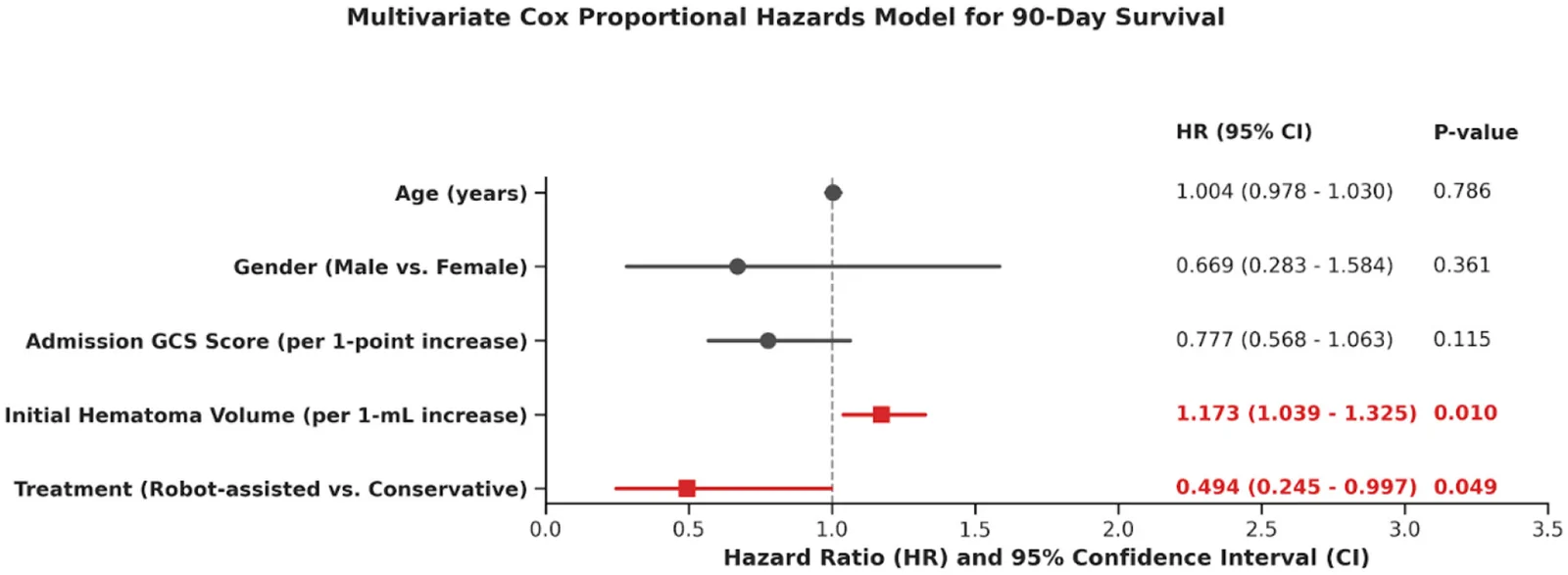
Multivariate cox proportional hazards model for 90-day survival (Forest plot).

### Propensity score-matched sensitivity analysis

To determine whether the survival benefit observed in the full cohort could be explained by baseline imbalances between the treatment groups, we repeated the primary analyses in the propensity score-matched cohort.

After 1:1 propensity score matching, 34 matched pairs of patients with highly balanced baseline characteristics were included (34 patients in the robot-assisted aspiration group and 34 in the conservative treatment group; 68 in total; Table 3). Between-group balance was achieved for age (51.2 ± 11.8 vs. 51.5 ± 13.3 years, *p* = 0.916), admission GCS score (4.15 ± 1.60 vs. 3.88 ± 1.34, *p* = 0.462), initial hematoma volume (8.24 ± 2.93 vs. 8.24 ± 2.50 mL, *p* = 0.993), and the distribution of hemorrhage location (p = 0.822) (all *p* > 0.05). No statistically significant differences were observed between the two groups in the incidence of pneumonia (76.47% vs. 73.53%), stress ulcer (29.41% vs. 26.47%), rebleeding (8.82% vs. 5.88%), or intracranial infection (0.00% vs. 2.94%) (all *p* > 0.05).

**Table 3:** Baseline characteristics and complication rates in the propensity score-matched cohort.

Variable	Conservative group (*n* = 34)	Aspiration group (*n* = 34)	*p*-value
Baseline characteristics			
Age, years, mean ± SD	51.5 ± 13.3	51.2 ± 11.8	0.916
Male sex, *n* (%)	30 (88.2)	24 (70.6)	0.134
Admission GCS score, mean ± SD	3.88 ± 1.34	4.15 ± 1.60	0.462
Initial hematoma volume, mL, mean ± SD	8.24 ± 2.50	8.24 ± 2.93	0.993
Hemorrhage location, *n* (%)			0.822
Pons	24 (70.6)	24 (70.6)	
Pons + midbrain	9 (26.5)	8 (23.5)	
Midbrain only	1 (2.9)	2 (5.9)	
Complications and adverse events			
Rebleeding, *n* (%)	2 (5.9)	3 (8.8)	1.000
Intracranial infection, *n* (%)	1 (2.9)	0 (0.0)	1.000
Pneumonia, *n* (%)	25 (73.5)	26 (76.5)	0.779
Stress ulcer, *n* (%)	9 (26.5)	10 (29.4)	0.787

GCS, Glasgow Coma Scale; PSM, propensity score matching; SD, standard deviation.

In the matched cohort, all core volume-stratified outcome measures fully reproduced the findings obtained in the full cohort (Table 4, Figures 8, 9).

**Table 4:** Volume-stratified outcomes in the propensity score-matched cohort.

Outcome	5–10 mL subgroup (*n* = 54)			>10 mL subgroup (*n* = 14)		
	Conservative group (*n* = 28)	Aspiration group (*n* = 26)	*p*-value	Conservative group (*n* = 6)	Aspiration group (*n* = 8)	*p*-value
Change in GCS score from admission to discharge, mean ± SD	+0.21 ± 2.13	+0.88 ± 1.90	—	−0.17 ± 0.41	+0.62 ± 1.77	—
30-day survival, % (n/N)	35.7 (10/28)	80.8 (21/26)	0.001	33.3 (2/6)	37.5 (3/8)	1.000
90-day survival, % (n/N)	32.1 (9/28)	73.1 (19/26)	0.003	33.3 (2/6)	37.5 (3/8)	1.000
Distribution of 90-day mRS grade, *n* (%)			0.034			0.938
mRS 2 (slight disability)	0 (0.0)	2 (7.7)	—	0 (0.0)	0 (0.0)	—
mRS 3 (moderate disability)	4 (14.3)	1 (3.8)	—	0 (0.0)	0 (0.0)	—
mRS 4 (moderately severe disability)	2 (7.1)	5 (19.2)	—	0 (0.0)	0 (0.0)	—
mRS 5 (severe disability, bedbound)	3 (10.7)	10 (38.5)	—	2 (33.3)	3 (37.5)	—
mRS 6 (death)	19 (67.9)	8 (30.8)	—	4 (66.7)	5 (62.5)	—

GCS, Glasgow Coma Scale; mRS, modified Rankin Scale; PSM, propensity score matching.

**Figure 8:**
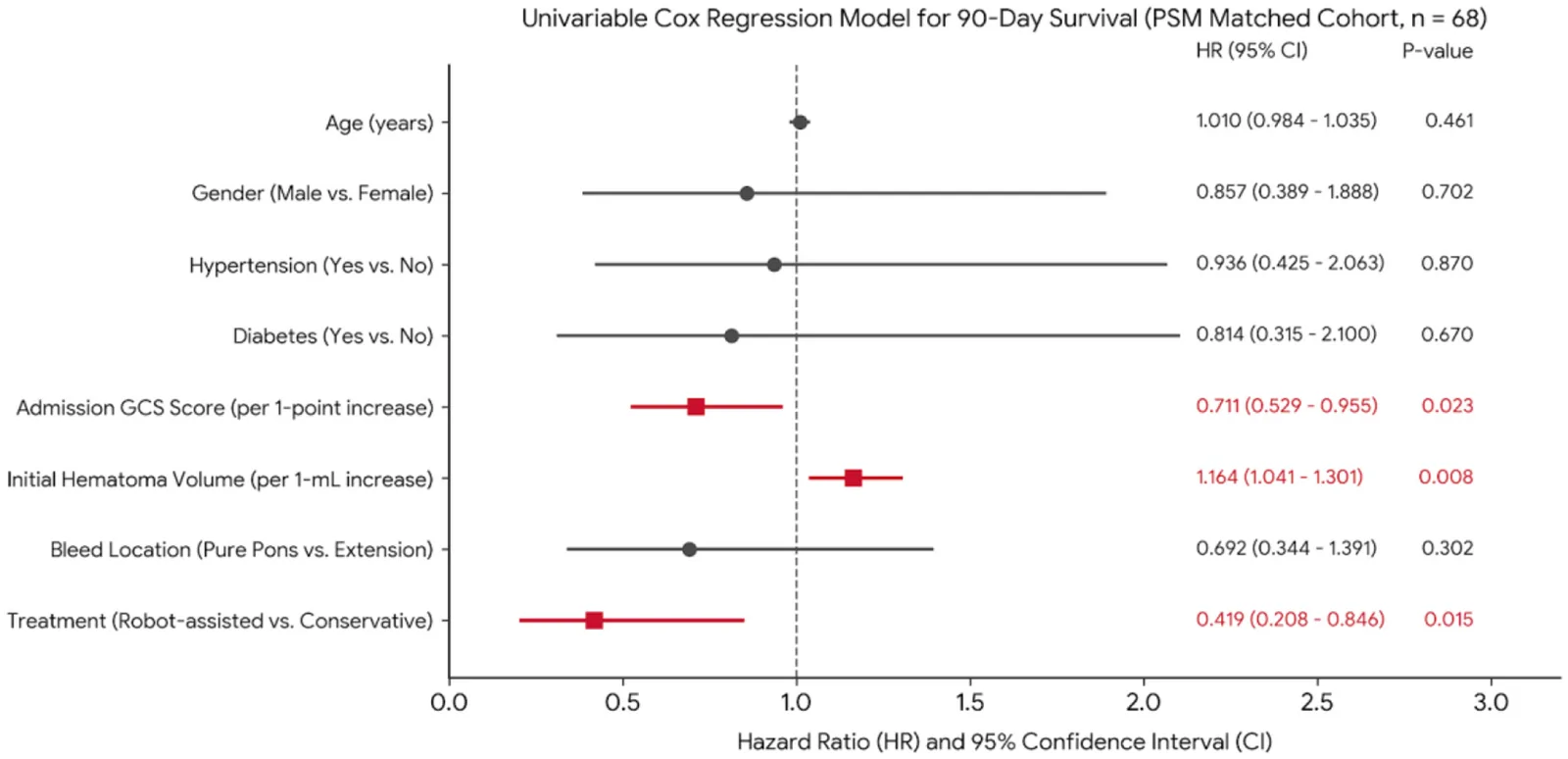
Univariable cox regression model for 90-day survival of critical PBSH patients (Forest plot, PSM matched cohort, *n* = 68).

**Figure 9:**
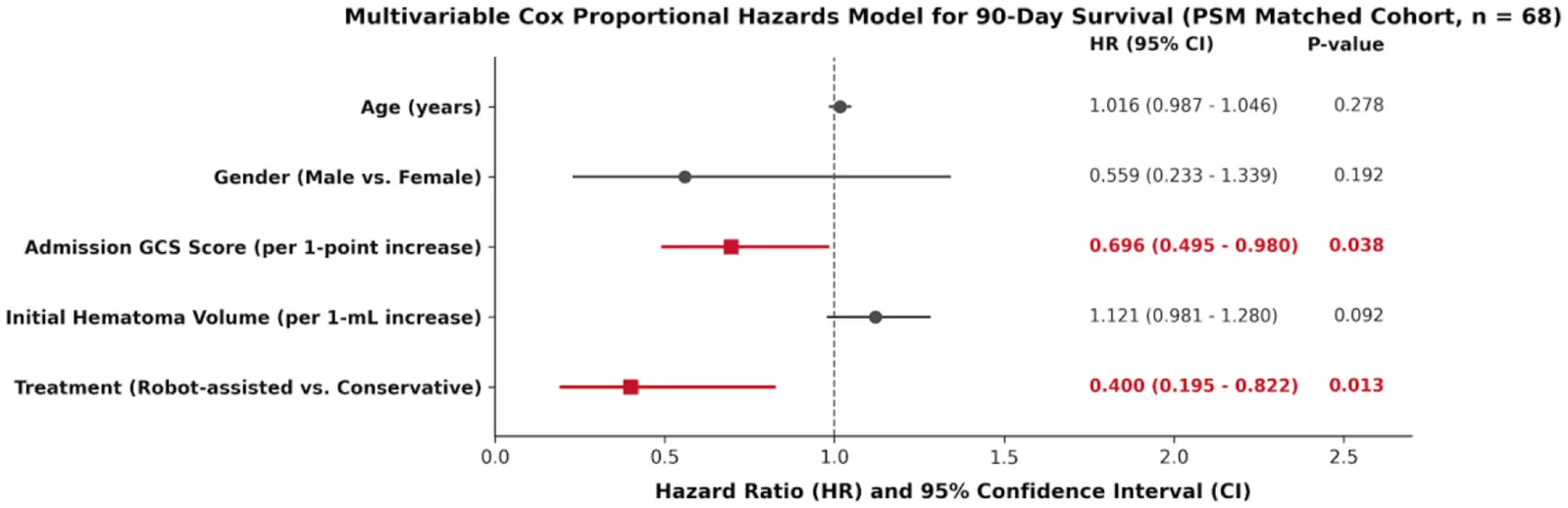
Multivariable cox proportional hazards model for 90-day survival (Forest plot, PSM matched cohort, *n* = 68).

## Typical cases

### Case 1

A 54-year-old female patient with a 10-year history of hypertension and no prior diabetes mellitus presented with 1 day of sudden unconsciousness. On admission, her GCS score was 3T with endotracheal intubation. Preoperative emergency cranial CT scans were imported into a robotic workstation for three-dimensional reconstruction, confirming a brainstem hematoma with a volume of 6.2 mL. The patient received robot-assisted stereotactic puncture and drainage for hematoma evacuation. A 24-h postoperative cranial CT scan confirmed accurate placement of the drainage catheter, with the residual hematoma volume reduced to 4.0 mL and marked resolution of mass effect. The patient regained partial spontaneous breathing on postoperative day 2, indicating effective alleviation of brainstem compression by early minimally invasive decompression. During hospitalization, her neurological function improved progressively. Endotracheal intubation was removed on postoperative day 7, with complete recovery of consciousness and spontaneous breathing. The patient was discharged with a GCS score of 11 and stable vital signs, without severe perioperative complications, including rebleeding, intracranial infection, and severe pneumonia. The patient survived at the 30-day follow-up with stable neurological status and no hemorrhagic recurrence or secondary neurological deterioration. At the 90-day follow-up, the patient exhibited continuous functional recovery, presenting only mild contralateral limb weakness without consciousness disturbance or cognitive dysfunction. The 90-day mRS score was 3, indicating moderate disability with partial independence in daily living, which demonstrated favorable long-term functional outcomes after robot-assisted minimally invasive treatment (Figure 10).

**Figure 10:**
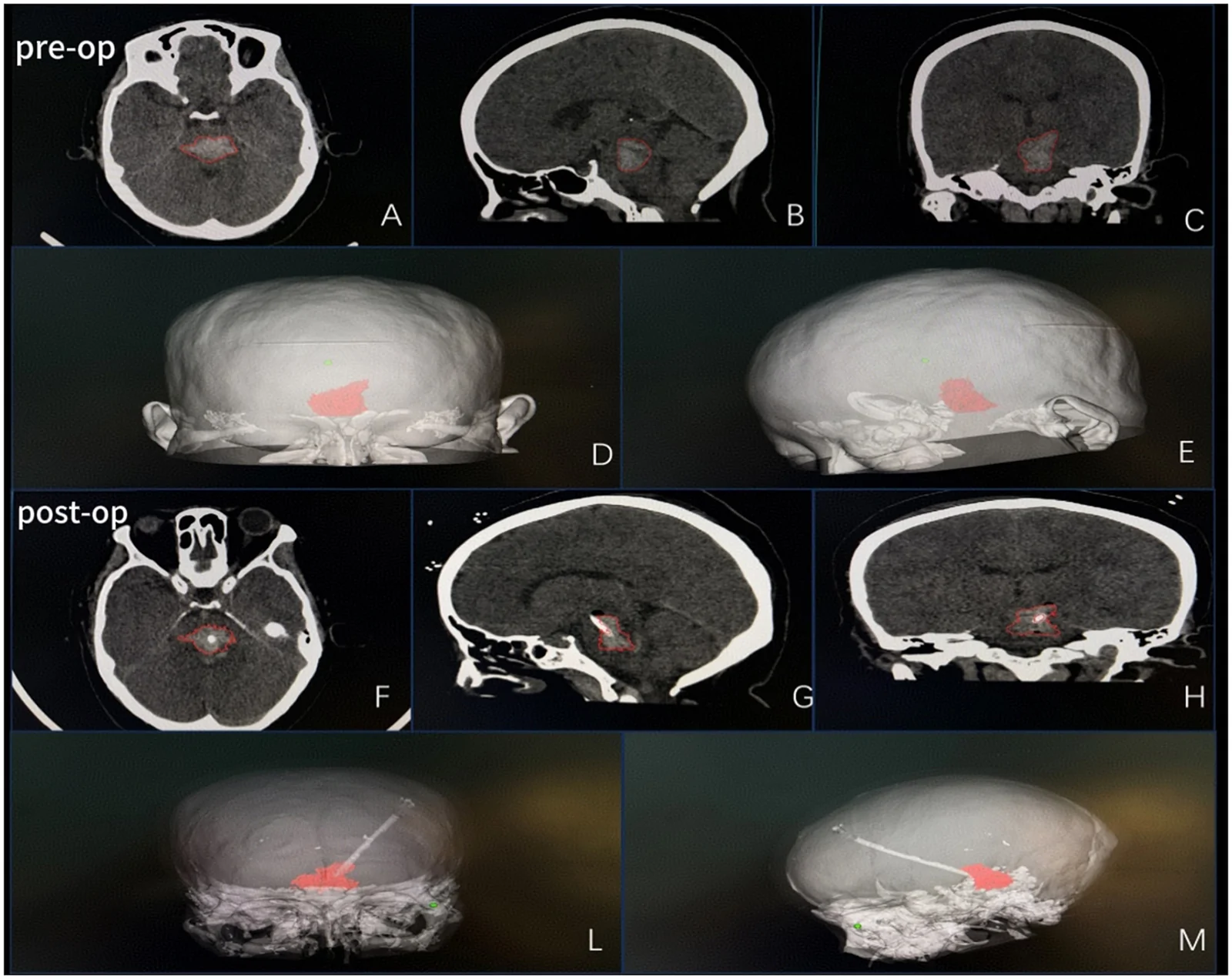
Imaging alterations of robot-assisted stereotactic drainage for critical PBSH. (Case 1) **(A–C)** Preoperative cranial CT imaging. **(D, E)** Three-dimensional robotic reconstruction of intracranial hematoma. **(F–H)** Postoperative follow-up CT imaging after robotic puncture and drainage. **(I, J)** Postoperative three-dimensional robotic reconstruction.

### Case 2

A 44-year-old male patient with a 30-year history of hypertension and no history of diabetes mellitus was admitted due to a 2-day duration of sudden unconsciousness. His admission GCS score was 3. Preoperative emergency cranial CT reconstruction identified an isolated brainstem hematoma with a volume of approximately 6 mL. After thorough communication and full informed consent from the patient's family, standardized conservative medical treatment was implemented. On hospital day 7, the patient presented occasional spontaneous eye opening, and follow-up cranial CT revealed gradual hematoma resorption with reduced lesion volume and decreased hematoma density. By hospital day 21, the patient was able to complete simple command-following movements of the right limb, and repeat CT scan demonstrated near-complete absorption of the brainstem hematoma. The patient was discharged on hospital day 21 to receive systematic rehabilitation therapy. At the 90-day follow-up, the patient achieved partial neurological functional recovery with a final mRS score of 3, presenting moderate disability and partial independence in daily activities (Figure 11).

**Figure 11:**
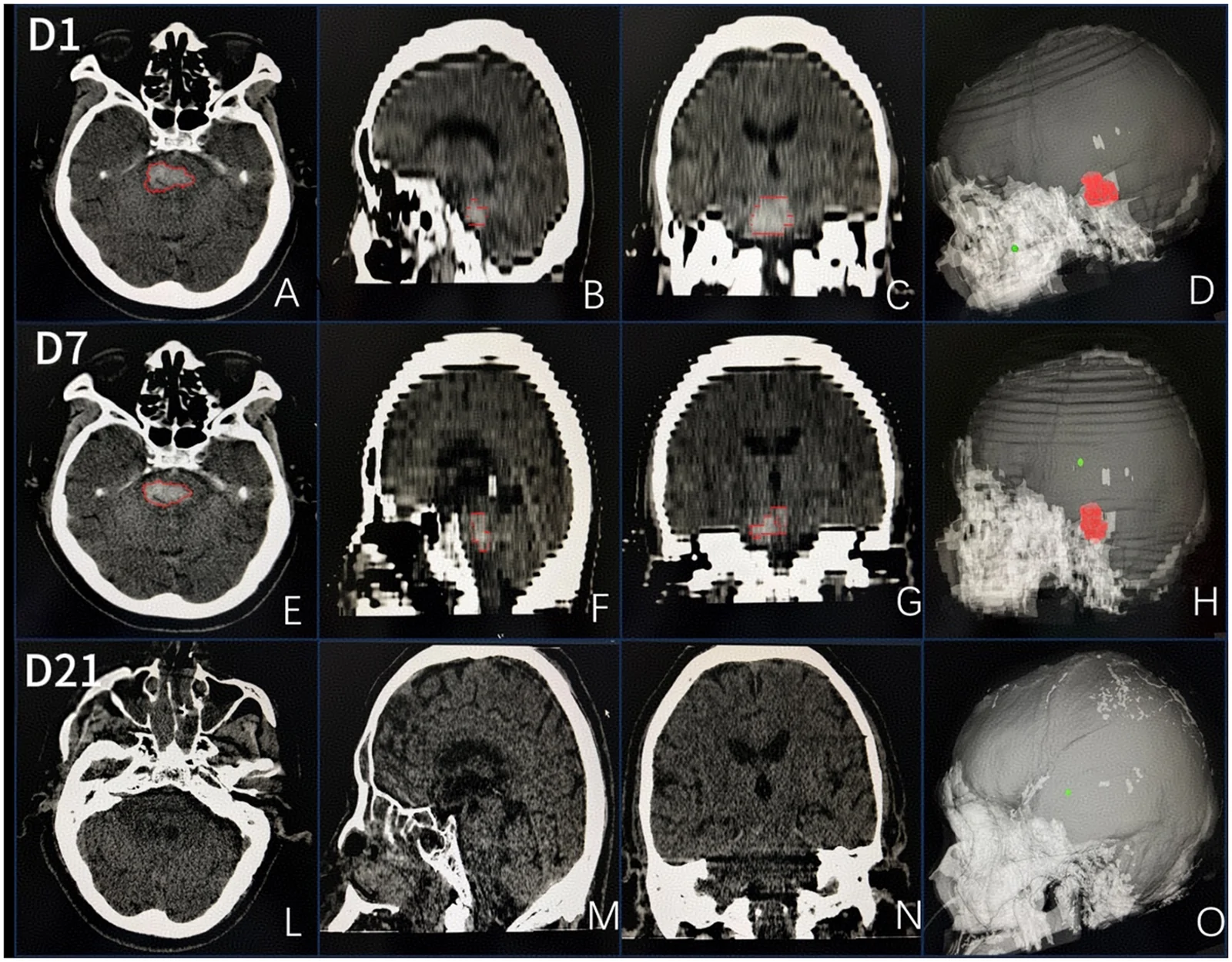
Imaging alterations of conservative treatment for critical PBSH. (Case 2) **(A–C)** Admission day 1 cranial CT scans. **(D)** Robotic three-dimensional reconstruction displays the initial spatial distribution and volume of the brainstem hematoma. **(E–G)** Day 7 follow-up CT images after conservative treatment. **(H)** Day 7 robotic reconstruction confirms decreased hematoma volume. **(I–K)** Day 21 follow-up CT scans reveal near-complete hematoma absorption. **(L)** Day 21 robotic reconstruction.

## Discussion

Safety assessment is the foremost prerequisite for any surgical intervention in the brainstem. The present study demonstrated that there were no statistically significant differences between the robot-assisted aspiration group and the conservative treatment group in terms of postoperative rebleeding rate or intracranial infection rate (both *p*> 0.05). Similarly, the incidences of perioperative pneumonia and stress ulcer were comparable between the two groups (*p*> 0.05). These findings strongly confirm that early robot-assisted puncture carries a favorable safety profile. The above data indicate that modern precision minimally invasive surgery can achieve effective physical decompression of the vital brainstem centers without incurring additional surgical risks, thereby establishing its reliable surgical feasibility (9, 10).

Multivariable Cox regression analysis confirmed that, after adjusting for age, sex, and admission GCS score, initial hematoma volume was a significant independent risk factor for 90-day mortality (11); each 1ml increase in hematoma volume independently raised the 90-day mortality risk by 17.3%. Conversely, early robot-assisted surgery emerged as a significant independent protective factor for 90-day survival, reducing the overall mortality risk by 50.6% (6). This protective effect exhibited a high degree of stratification specificity in the 5–10 ml hematoma subgroup, not only substantially lowering the 30-day and 90-day mortality rates (12), but also leading to a significant improvement in GCS score at discharge, which is of great importance for early neurological recovery and salvage.

Stratified analysis based on initial hematoma volume revealed distinctly different surgical benefit windows. For the 5–10 ml range, the 90-day Kaplan–Meier cumulative survival curves and log-rank test in this study provided robust statistical evidence of survival benefit (*p* = 0.010), confirming this range as the “golden window” for robot-assisted puncture. Precise volume reduction effectively interrupted the fatal compression of the vital brainstem centers caused by acute mass effect and secondary edema (6, 12, 13). For hematomas exceeding 10 ml, our data are insufficient to determine whether robot-assisted aspiration is beneficial. In the unmatched cohort, 30-day survival was 60.0% (6/10) vs. 33.3% (2/6) — an absolute difference of +26.7 percentage points — but the 95% confidence interval (−19.9 to +59.8) spans both appreciable benefit and appreciable harm, and in the propensity score-matched cohort the same comparison gave 37.5% (3/8) vs. 33.3% (2/6), a difference of only +4.2 percentage points. The apparent short-term advantage was therefore not reproduced after baseline characteristics were balanced, which suggests it was substantially influenced by between-group differences at baseline; the 90-day survival curves converged, and all survivors in both arms were severely disabled (mRS grade 5). One interpretation is that the high-pressure arterial flow generated instantaneously by massive hematomas causes devastating and irreversible mechanical destruction of the ascending reticular activating system and of vital brainstem nuclei that cannot be reversed by physical drainage alone (5, 14). however, with 16 patients this explanation cannot be distinguished from a simple loss of statistical power, and we present it as a hypothesis to be tested rather than as a conclusion supported by our data. Traditional pooled analyses tend to obscure the true surgical benefit (15); only through rigorous volume stratification can the statistical dilution of moderate-volume hematoma benefit data by extreme critical cases be avoided, which holds important discriminative value for guiding precise, stratified clinical decision-making.

The marked improvement in early survival rates coupled with the lack of substantial improvement in 90-day functional outcomes in the aspiration group reflects the irreversibility of primary neurological damage in critical PBSH (16, 17). Even though minimally invasive catheter drainage can maximally evacuate the hematoma mass and salvage the ischemic penumbra, surviving patients inevitably exhibit severe disability at 3 months post-onset (18, 19). A gain in survival that is not accompanied by a gain in independence cannot, however, simply be equated with benefit. Whether survival with severe disability requiring continuous care is preferable to death is a value judgment that our data cannot settle: some patients and families regard it as unacceptable, others as preferable to death, and preferences differ substantially between individuals, families and cultural settings. We therefore caution explicitly against reading the reduction in 90-day mortality as evidence of improved quality of life (20). Our findings are consistent with those of Hong and colleagues, whose systematic review and meta-analysis of robot-assisted surgery for primary brainstem hemorrhage likewise found that the survival advantage of the procedure is not matched by a comparable improvement in functional outcome; that analysis, like ours, was limited by small, retrospective, non-randomized primary studies with heterogeneous and short follow-up. Accordingly, the decision to operate in this population should be made through shared decision-making in which the realistic probability of survival with severe disability — not merely the probability of survival — is communicated explicitly, and in which best supportive and palliative care is presented on equal terms. Future trials and registries in this field should adopt functional independence, health-related quality of life and, ideally, patient- or proxy-reported outcome measures as co-primary endpoints, because mortality alone will systematically overstate the value of an intervention that converts death into severe dependency.

Several limitations of this study should be acknowledged. Selection bias is inherent in any retrospective, non-randomized comparison, and it is the principal caveat attaching to our findings. Treatment allocation was determined by the attending neurosurgeon in consultation with the patient's family rather than by randomization. Propensity score matching balanced the measured baseline characteristics, and the survival advantage persisted in the matched cohort; neither matching nor multivariable adjustment can, however, address factors that were not recorded. Such unmeasured clinical and psychosocial factors may include the rate of evolution of secondary peri-hematomal edema, the fragility of the deep microvasculature, and the willingness and degree of commitment of the patients' families with regard to the intensity of intensive care and to early tracheostomy and ventilator-weaning care. Each of these could plausibly have influenced both the decision to operate and the probability of death, and each may therefore have contributed residual confounding to the observed survival trajectories. The matching procedure itself also entailed a loss of 11 patients, from 79 to 68, and it is for this reason that the full-sample multivariable Cox model was retained as the primary analysis and the matched cohort used as a sensitivity analysis — an arrangement that reconciles adequate statistical power with the minimization of selection bias.

A second limitation is the modest sample size, which bears most heavily on the >10 mL stratum. That stratum contained only 16 patients, and only 14 after matching. For 30-day survival the absolute difference was +26.7 percentage points (95% CI: −19.9 to +59.8); the width of this interval is more informative than the *post-hoc* power of 0.28, because it shows the data to be compatible with a large survival gain, with no difference, and with clinically meaningful harm. Detecting a difference of this magnitude at α = 0.05 with 80% power would require approximately 60 patients per group, that is about 120 in this stratum alone, more than seven times the number enrolled here. In the matched cohort the same comparison gave 37.5% (3/8) vs. 33.3% (2/6), a difference of only +4.2 percentage points (95% CI: −39.6 to +43.9), so the point estimate observed in the unmatched cohort was not reproduced. We therefore regard the >10 mL stratum as inconclusive and exploratory: it provides no evidence of benefit and equally no evidence of futility, and it should not be cited in either direction.

The assessment of functional outcome was limited in two respects. First, follow-up was censored at 90 days, which is short for brainstem injury: neurological recovery, central compensation and rehabilitation gains frequently continue for 6 to 12 months or longer, and disability measured at 3 months may overstate permanent dependency. We do not assume that a longer window would necessarily favor either treatment arm, since it could reveal either further recovery or late deterioration and complications such as shunt-dependent hydrocephalus, or the longer-term impact of tracheostomy and gastrostomy dependence, which are highly relevant to how patients and families weigh the trade-off between survival and severe disability. Second, functional outcome is reported descriptively by mRS grade: although the overall distribution of mRS grades was compared between groups, we did not perform a formal comparison of the proportion of patients free of severe disability or death, nor an ordinal (proportional-odds) shift analysis yielding a common odds ratio. Identifying the minority of patients who can achieve independence after critical PBSH is a priority that our sample is too small to address.

Finally, all participating centers were located within a single province, and the findings may not generalize to settings with different surgical volumes, operator experience or end-of-life care practices. Prospective multicenter studies with larger cohorts, extended follow-up beyond one year incorporating standardized cognitive and quality-of-life evaluations, and finer hematoma volume stratification are warranted to validate these findings, refine surgical indications and further establish the therapeutic window for robot-assisted intervention in critical PBSH.

## Conclusions

1. Robot-assisted stereotactic puncture and drainage for critical PBSH demonstrates a favorable safety profile, without increasing the risks of rebleeding, intracranial infection, pneumonia, or stress ulcer.
2. Early minimally invasive aspiration was associated with higher 30-day survival and facilitated early recovery of consciousness; the 90-day survival benefit was statistically significant in the 5–10 mL stratum (*n* = 63) but not in the overall cohort, and the >10 mL stratum (*n* = 16) was underpowered and inconclusive.
3. Initial hematoma volume is an independent risk factor for 90-day mortality in patients with PBSH, whereas robot-assisted puncture was independently associated with lower 90-day mortality. This survival advantage was not accompanied by a demonstrated improvement in 90-day functional outcome, and longer follow-up with standardized quality-of-life assessment is required to determine whether it translates into a net benefit for patients.

## Data Availability

The datasets generated and analyzed during the current study are not publicly available due to patient privacy regulations but are available from the corresponding author on reasonable request.

## References

[1] Morotti A Jessel MJ Brouwers HB Falcone GJ Schwab K Ayres AM . CT angiography spot sign, hematoma expansion, and outcome in primary pontine intracerebral hemorrhage. Neurocrit Care. (2016) 25:79–85. doi: 10.1007/s12028-016-0241-2 26759226 PMC4940347

[2] Chen D Tang Y Nie H Zhang P Wang W Dong Q . Primary brainstem hemorrhage: a review of prognostic factors and surgical management. Front Neurol. (2021) 12:727962. doi: 10.3389/fneur.2021.727962 34566872 PMC8460873

[3] Ding Y Qi M Zhang X Dong J Wu D. Stereotactic hematoma puncture and drainage for primary pontine hemorrhage: clinical outcomes and predictive model. Heliyon. (2024) 10:e27487. doi: 10.1016/j.heliyon.2024.e27487 38486743 PMC10938131

[4] Tang Z Huang W Chen Q Guo C Zheng K Wei W . Curative effect analysis of robot-assisted drainage surgery in treatment of spontaneous hypertensive brainstem hemorrhage. Front Neurol. (2024) 15:1352949. doi: 10.3389/fneur.2024.1352949 38469591 PMC10925720

[5] Sun X Zhu J Lu M Zhang Z Li C Zhan R. Robot-assisted puncture versus conservative treatment for severe brainstem hemorrhage: clinical outcomes comparison with experience of 138 cases in a single medical center. World J Emerg Surg. (2025) 20:15. doi: 10.1186/s13017-025-00592-9 40001232 PMC11854005

[6] Pustilnik HN Fontes JHM Porto Junior S Punukollu A Sousa MP Da Cunha BLB . Stereotactic aspiration versus conservative management for primary brainstem hemorrhage: a systematic review and meta-analysis. Clin Neurol Neurosurg. (2024) 246:108583. doi: 10.1016/j.clineuro.2024.108583 39395282

[7] Tian D Liu L Chen X Liu Q. Successful puncture and drainage of primary brainstem hemorrhage with robotic assistance. J Craniofac Surg. (2026) 37:e176–8. doi: 10.1097/SCS.0000000000012068 41081559

[8] Yu Z Zhang X Xu Q Zhang Z Xia Y Li H . Effect of hematoma volume on the 30-day mortality rate of patients with primary hypertensive brainstem hemorrhage: a retrospective cohort study. Front Surg. (2023) 10:1136296. doi: 10.3389/fsurg.2023.1136296 37215349 PMC10196262

[9] Almohammedi RM Almutairi H Alhoussien RO Alotayan MT Almutairi AK Bafail WO . Brainstem hemorrhage is uncommon and is associated with high morbidity, mortality, and prolonged hospitalization. Neurosciences. (2020) 25:91–6. doi: 10.17712/nsj.2020.2.20190102 32351245 PMC8015522

[10] Du L Wang J Li C Gao B. Effects of stereotactic aspiration on brainstem hemorrhage in a case series. Front Surg. (2022) 9:945905. doi: 10.3389/fsurg.2022.945905 36061043 PMC9437539

[11] Ding Y Xu Y Wang Y Dong J. A nomogram risk prediction model for poor outcome of primary brainstem hemorrhage based on clinical data and radiographic features. Neurol Sci. (2023) 44:3967–78. doi: 10.1007/s10072-023-06866-x 37213039

[12] Zhou J Wang R Mao J Gu Y Shao A Liu F . Prognostic models for survival and consciousness in patients with primary brainstem hemorrhage. Front Neurol. (2023) 14:1126585. doi: 10.3389/fneur.2023.1126585 36908614 PMC9995821

[13] Huang K Ji Z Sun L Gao X Lin S Liu T . Development and validation of a grading scale for primary pontine hemorrhage. Stroke. (2017) 48:63–9. doi: 10.1161/STROKEAHA.116.015326 27932606

[14] Xu C He W Yi T Zhang H Xu J Ma J. Robotic frameless stereotactic aspiration with thrombolysis for primary pontine hemorrhage: a therapeutic evaluation of a retrospective cohort study. J Neurol Surg A Cent Eur Neurosurg. (2025) 86:111–9. doi: 10.1055/a-2235-5453 38151032

[15] Zhang S Chen T Han B Zhu W. A retrospective study of puncture and drainage for primary brainstem hemorrhage with the assistance of a surgical robot. Neurologist. (2023) 28:73–9. doi: 10.1097/NRL.0000000000000445 35593907

[16] Wei M Chen Q Yang X Zhu X Tian X Tong Q . 3-dimensional technology-assisted minimally invasive surgery for the treatment of primary brainstem hemorrhage: a prospective cohort study. World Neurosurg. (2025) 194:123487. doi: 10.1016/j.wneu.2024.11.070 39579926

[17] Cai C Yan C Chen S Yang W Huang Y Ma J . Development and validation of a prediction model for 30-day mortality and functional outcome in patients with primary brainstem hemorrhage. Cerebrovasc Dis. (2024) 53:79–87. doi: 10.1159/000530348 37231825

[18] Wei S Gu L Fan Y Ji P Yang L Li F . Development and validation of a prognostic nomogram for predicting outcomes in brainstem hemorrhage patients. Sci Rep. (2025) 15:1300. doi: 10.1038/s41598-024-80264-x 39779767 PMC11711512

[19] Meng X Wang Q Pei X Xie F. Prognosis and influencing factors of early microsurgery for severe hypertensive brainstem hemorrhage. Dis Markers. (2022) 2022:5062591. doi: 10.1155/2022/5062591 36193500 PMC9526571

[20] Hong X Li Y Xu T Han K Hou L. Robot-assisted surgery for primary brainstem hemorrhage: a systematic review and meta-analysis. Brain Hemorrhages. (2025) 6:331–8. doi: 10.1016/j.hest.2025.11.001

